# Intimate Partner Violence against Women with Disabilities in Spain: A Public Health Problem

**DOI:** 10.3390/ijerph18020728

**Published:** 2021-01-15

**Authors:** María-Leticia Meseguer-Santamaría, Francisco Sánchez-Alberola, Manuel Vargas-Vargas

**Affiliations:** Faculty of Economics and Business Administration, University of Castilla-La Mancha, 02071 Albacete, Spain; francisco.salberola@uclm.es (F.S.-A.); manuel.vargas@uclm.es (M.V.-V.)

**Keywords:** disability, gender, intimate partner violence, public health, Spain, women

## Abstract

Violence against women with disabilities is a social problem with important consequences for their physical and mental health. The World Health Organization (WHO) declared violence against women as a public health priority issue in 1996 and the fact that violence is used by the intimate partner and upon women with disabilities exacerbates the situation. Therefore, this is an issue that must be addressed from a public health viewpoint. Violence is studied from various aspects: Physical, psychological, sexual, or social control, and its multiple consequences in women’s health and the use of health services. In this perspective, with the data from the VI Violence against Women Macro-survey 2019 (VWM-2019) and adjusted to Spain, this study examines the incidence of intimate partner violence and its consequences in the health of women with disabilities and its impact on health services. Using binary logistic regression, the greater vulnerability of this group to these attacks is stated and the need to address this issue to improve the health of these people is brought to light.

## 1. Introduction

Violence against women is a social issue that implies serious consequences for the health of the people who experience it. In its resolution 48/104 of 20 December 1993, the United Nations General Assembly establishes that violence against women means “any act of gender-based violence that results in, or is likely to result in, physical, sexual or psychological harm or suffering to women, including threats of such acts, coercion or arbitrary deprivation of liberty, whether occurring in public or in private life” [1], and recognizes the urgent need to act on it, affirming that this type of violence constitutes a violation of human rights and fundamental freedoms. Following this discourse and considering the extension and severity of this problem, the World Health Organization (WHO), an organization that is in charge of coordinating international efforts on public health, declared, in 1996, violence against women as a public health priority issue, and recognized the significant consequences, immediate and future, physical and psychological [2].

In the last decades, social concern about abuse of women has increased. National and international organizations have begun to develop studies and surveys to collect essential data in order to make scientific investigation more efficient and bring visibility to this problem. In this context, we find the VI Violence against Women Macro-survey 2019 (VWM-2019) [3], which provides information about the Spanish case.

This kind of violence has profound social and cultural roots and means a great imbalance in gender relations [4]. Studies about the prevalence of violence on women show that, in Europe, 8% of women have suffered physical or sexual violence in the last year; one third have suffered some type of physical or sexual aggression since the age of 15; 32% have suffered psychological violence from their intimate partner; 5% have suffered economic violence from their current partner; and 13% have suffered economic violence in previous relationships, as shown by the European Union Agency for Fundamental Rights (FRA) [5]. At the Spanish level, taking into account the VWM-2019 figures, intimate partner violence affects 25% of women.

When it is the intimate partner (current or former) who uses violence, the situation gets worse, since the characteristic of intimacy is added, and invisibility increases. The loneliness of the victim increases. Abused women have more health problems, both physical and psychological, and make greater use of health services. Diseases like depression, Post-Traumatic Stress Disorder, or alcohol and drug addiction have a higher incidence in women who are victims of Intimate Partner Violence (IPV) [6]. Moreover, it is the most common form of violence suffered by women [4]. IPV is physical, psychological, and sexual violence, or economic or social control against women by the person who is or has been their spouse.

Disabilities of women experiencing IPV are in many cases an aggravating factor in this situation; their greater social isolation makes them more dependent on their partner, and therefore, more vulnerable to such violence and with more difficulties to seek help and get out of the situation. Thus, it is essential to analyze the condition of disability as a factor that increases the prevalence of women who suffer violence from their partners. Moreover, it also represents a condition which increases the worsening of these women’s health and their use of health services.

Given their greater vulnerability, women with disabilities suffer a higher impact of the IPV (physical, psychological, and sexual) [7,8,9,10]. These violent displays are usually more severe and aggravated due to specific factors: Social isolation and discrimination [11]; stigmatization and its consequences on women with disabilities and their self-esteem [12,13]; how social and sexual stereotypes increase partner abuse and aggression [14,15]; deal with limitations, both physical and attitudinal, on access to social and health resources which lead to a greater isolation [16]; or the incidence of economic factors, such as a lower socioeconomic level or higher rates of unemployment and poverty [15,17].

In the case of the United States, the incidence of violence in different areas and, in particular, IPV is up to three times higher as regards women with disabilities [9,18]. This fact is evident not only in direct physical consequences (injuries, lesions, etc.): IPV affects a worse general health state [19], and it impacts on mental health problems such as anxiety, depression, post-traumatic stress, sleep disorder, etc. [20,21].

Our study focuses on Spain and analyzes the data from the VI Violence against Women Macro-survey 2019 carried out by the Center for Sociological Research (CIS). Some researchers analyze the Spanish situation, making use of the different waves of said survey, identifying it as one of the preferred quantitative sources for Spain [22,23,24].

Together with the gender perspective, this analysis focuses on women with disabilities and tries to analyze the relation between violence from their partners (current or former) and the health and health care consequences for these women.

Therefore, the following hypotheses are established:

**Hypotheses 1** **(H1).** *Disability increases the prevalence of IPV in Spanish women*.

**Hypotheses 2** **(H2).** *Disability increases the prevalence of health problems and the use of health services in Spanish women who have experienced IPV*.

## 2. Materials and Methods

### 2.1. Violence against Women Macro-Survey 2019

The VWM-2019 was carried out from 12 September 2019 to 1 October 2019, and was addressed to the female population aged 16 and over residing in Spain. The sampling procedure was stratified by a multi-stage conglomerate selecting the primary sampling units (municipalities) and the observation units (individuals) based on random routes and age and occupation quotas. The questionnaires were carried out through Computer-Assisted Personal Interviews (CAPI) at home. A sample size of 10,000 interviews was designed, and 9568 interviews were conducted in a total of 582 municipalities and 52 provinces. Regarding the sample error, for a confidence level of 95.5 (two-sigma), and P = Q, the absolute error is ±1% for the whole of the sample and on the assumption of a simple random sampling. The objectives of this survey are to estimate the prevalence of violence against women in Spain, differentiating between partner, former partners, and an outside party; to identify the types of violence suffered: physical, sexual, psychological, emotional, control, and economic, and to measure their frequency and severity; to determine the effects of violence against women on their health as well as the impact on their working life. It also analyzes the formal and informal support received and the impact on the children of the victims [3].

The “International Classification of Functioning, Disability and Health” (ICF), defines disability as a “generic term encompassing impairments, activity limitations and participation restrictions” [25]. At the European level, based on the ICF, understood in a broad basis, several European institutions have agreed on an operational statistical definition that allows international comparison. It is about considering that persons with disabilities are those “persons who have had limitations in basic daily life activities due to health problems for at least the last 6 months”. This measure, known as the Global Activity Limitation Index (GALI), belongs to the Minimum European Health Module (MEHM) and is present in some of the major European surveys, such as the European Health Interview Survey (EHIS) or the Statistics on Income and Living Conditions (SILC), in OECD studies. Additionally in the Spanish case, there is an administrative “certificate of disability”, which recognizes degrees of disability, and provides legal benefits to people who have a degree of disability of at least 33%. However, this certificate, based on medical criteria, is more restrictive than the bio-psycho-social model of disability adopted by the ICF.

In this statistical operation, it is possible to identify women with disabilities using their answers to two questions: Having the certificate of disability with a degree equal to or greater than 33% (variable M0P8), and a self-classification item related to the limitations on their daily activity: “Activity limitation and suffering from some ailment, lesion or illness that has lasted or will last more than 1 year” (variable M0P9), whose possible answers are: “No”; “Yes, slightly”; and “Yes, severely”. Thus, those found in the latter case are also considered as women with disabilities.

According with the bio-psycho-social model of disability, both criteria are used in this study, and people with disabilities are identified as those who manifest being severely disabled, within the scope of the question M0P9 above, and also those who certify that they have a 33% or more disability, in the scope of question M0P8.

Intimate Partner Violence is addressed by different items related to different types of violence: Physical violence, psychological violence, from social control to economic control violence, sexual violence, and fear-producing in the victim violence are measured.

### 2.2. Socio-Demographic Profile of the Women in the Sampling

In order to know the socio-demographic profile of the Spanish women who experience IPV, the most relevant characteristics of the survey, the basis of this study, are analyzed. They are: Women with disabilities, Age, Educational Level, Labor status, Nationality, Currently having an intimate partner, Mother with minor children, and Town size.

All these variables are shown in Table 1, identifying the number of women in each group, the percentage they represent within each variable and, lastly, the percentage of them who experience or have experienced IPV. Considering the 9568 women who participated in the survey, 25% of them have suffered IPV [24]. Hence, comparing this percentage against the respective percentage of groups and variables, the more vulnerable groups can be identified.

The age range in which women experience more IPV is between 31 and 45 years old, with 31.70%. Women with higher education present a prevalence which decreases down to 18.8%. Regarding nationality, prevalence of women with only Spanish nationality is 23.20%, 36.50% for women with another nationality, and 40.80% for those with both Spanish and other nationality. The prevalence of IPV among women who currently have a partner shows a data of 22.70% compared to 33.90% of women who do not have a current partner. Mothers with minor children endure more IPV than those women who have no children, with a difference of 8 percentage points, from 29.9% to 21.50%. Finally, regarding the size of the town, a lower prevalence was observed in those municipalities with less than 10,000 inhabitants, 20.90%. The rest present values near 26%.

### 2.3. Quantitative Study (Method)

In keeping with other studies [26,27], the logistic regression technique was used. How IPV affects the health of the victims and the use they make of health services has been analyzed. Next, how the probability of experiencing IPV increases in women with disabilities has been estimated, differentiating the various types of violence. Then, the influence of disability on health and the use of health services has been measured, but limited to those women who have experienced IPV. Finally, for that same group, seven other variables referring to the direct consequences of IPV have been studied. In the multivariate statistical analysis tool used, the endogenous variable, or answer, is a dichotomous variable and the dependent variables can be quantitative or qualitative.

The statistical model expresses the probability of the reference category of the endogenous variable by the logistic transformation of a linear combination of the exogenous variables:

$$P(Y)=\frac{e^{\beta_{0}+\sum \beta_{i}X_{i}}}{1+e^{\beta_{0}+\sum \beta_{i}X_{i}}}$$

Equivalently, the model can express the odds ratio (OR) of the reference category as a linear regression on exogenous variables:

$$\ln\left(\frac{P(Y)}{1-P(Y)}\right)=\beta_{0}+\sum_{i=1}^{k}\beta_{i}X_{i}$$

In this case, as exogenous variables are dichotomous, the exponential value of their regression coefficient β is equal to the OR value.

Endogenous variables are:

- Disabilities: A dichotomous variable which takes the value 1 if the woman has some form of disability, and 0 if not.
- IPV: A dichotomous variable which takes the value 1 if the woman has experienced violence from an intimate partner, and 0 if not.

Three groups of explanatory variables have been considered:

- Health and use of health services; dichotomous variables which take the value 1 if the woman answers yes, and 0 if not.
  - Staying in bed at least one day for health reasons.
  - Visit a healthcare center or general practitioner.
  - Admission to a hospital.
  - Use of emergency service.
  - Visit to a psychologist, psychotherapist, or psychiatrist.
  - Consumption of tranquilizers.
  - Consumption of antidepressants.
  - Consumption of analgesics.
- Types of violence from the intimate partner; dichotomous variables which take the value 1 if the woman has experienced it, and 0 if not.
  - Control violence.
  - Economic violence.
  - Psychological violence.
  - Physical violence.
  - Sexual violence.
  - Fear.
  - All types of violence.
- Consequences arising from having experienced IPV; dichotomous variables which take the value 1 if the woman has experienced the consequence, and 0 if not.
  - Physical consequences due to IPV.
  - Need for healthcare due to IPV.
  - Psychological consequences due to IPV.
  - Medicine use due to IPV.
  - Alcohol use due to IPV.
  - Hard drugs use due to IPV.
  - Access to social services due to IPV.

The variable “Need for healthcare services due to IPV” is a recoding of the original survey question “Access to health services by episodes of physical or sexual violence of the intimate partner”. It is assigned the value “Yes” when she answers “Yes, I had to stay in the hospital”, “Yes, someone from the health services treated me”, and “No, but I should have received it”. The first two options are related to the actual use of health services, while the third includes the “need” for such services, which the woman thinks she should have received, but did not obtain (lack of reporting, fear of publicly assuming IPV, past inadequate responses from medical professionals, etc.).

The statistical software package that has been used is SPSS version 27.

## 3. Results

According to the VWM-2019, in Spain 25.04% of women have experienced any kind of IPV, with an incidence of 23.25% of psychological violence and of 14.24% of physical or sexual violence.

Regarding the first hypothesis (H1), data show that the issue of IPV against women is worse for women with disabilities, since they experience a greater incidence on all types of violence, as shown in Table 2:

Percentages of women who have experienced IPV are significantly higher in all types of violence for women with disabilities, as corresponding F-statistics indicate, all of them with *p*-value < 0.001.

In respect to control violence, 23.3% of women with disabilities suffer it, whereas only 17.1% of women without disabilities do. This means that disability multiplies the probability of suffering this kind of control by 1.478.

Regarding the second category, 7% of the female population without disabilities suffers economic violence. This number almost doubles, 13.1%, for the group of women with disabilities. This type of violence has a lower incidence in the female population. The corresponding OR of 2.008, the highest in the categories analyzed, shows that women with disabilities are twice as likely to suffer economic violence.

Psychological violence is the one that shows the highest percentages, both in women who have a disability and in those who do not, with 30.1% and 22.5%, respectively. Disability also increases the probability of suffering such violence by almost 50%.

In relation to the next category, physical violence, the odd ratio reaches 1.657, so that women with disabilities who suffer this violence are almost two thirds more than those women without disabilities. The percentage of the female population reporting this violence is 10.4% of women without disabilities and 16.1% of women with disabilities.

Sexual violence follows the same pattern: 8.4% of women among those without disabilities and 12.8% of women among those with disabilities suffer it. Thus, having disabilities means an increase of slightly more than 50% of suffering this type of violence.

The percentages of women who report having been afraid of their intimate partners reach 18.2% and 13.4%, according to whether the victims have a disability or not. In addition, with an odd ratio of 1.448, disability is a factor that multiplies by one and a half times the probability of feeling this fear.

Thus, disability is a significant factor that increases the probability of experiencing IPV, as indicated by the odd ratios discussed and summarized in Table 2.

It can thus be said that disability increases by around 50% the probability of experiencing almost all types of intimate partner violence, highlighting the case of economic violence, where this probability increases by a 100%.

Therefore, within the group of women who have experienced IPV, disability is a factor that increases the prevalence of health problems and the use of health services, as shown in Table 3 below:

For the three most general items (staying in bed at least one day for health reasons, visit to a healthcare center or general practitioner, and admission to a hospital), there is an increase due to disability in the level of use of health services by experiencing IPV, between three and six percentage points, with significance levels ranging from 5% to 10%. In almost all the remaining items, the increase in use of health services associated with disability is highly significant (*p*-value < 0.001), varying between 12% and 15%. Finally, for the consumption of analgesics, there is a slight decrease of 4%, not significant.

As shown in Table 3, disability is a factor that increases the likelihood of women who have experienced IPV using health services, thus showing the special attention that this group should receive due to the impact it has on the health system.

In addition to these global items available to all women in the sample, for the subgroup of those who have experienced IPV, the VWM-2019 provides items related to the direct consequences of this violence, as shown in Table 4:

Disability is a factor that significantly increases the prevalence of suffering physical consequences, raising the percentage to 32.2%, and, to a lesser extent, that of suffering psychological consequences, although the prevalence in this case is already quite high.

Additionally, highly significant are the increases in the prevalence of the need for health services, the use of medicines, and the need to resort to social services, which are 11.3%, 12.4%, and 6.4%, respectively. The variations in the prevalence of alcohol or hard drugs use are, by comparison, smaller and not statistically significant.

In general, as observed in Table 4, which shows the odds ratio of the impact of disability on the consequences of IPV, having a disability is a factor that increases the probability of having to resort to health or social services by more than 50%, 92.6% the probability of needing medicines, 37.4% the probability of suffering physical consequences, and 28.5% the probability of suffering psychological consequences.

## 4. Discussion

The results of the study reflect the impact of IPV on women’s health and the health system in Spain, confirming its study as a matter of Public Health, in line with the consideration made by the WHO in 1993.

The prevalence of women who have experienced IPV in Spain reaches 25.04%, similar to that obtained by WHO for Europe (25.4%), for America (29.8%), Western Pacific Region (24.6%), for Higher Income Countries (23.2%) [28], or for USA [9]. It is significantly lower than in the African Region (36.6%), Eastern Mediterranean Region (37.0%), or South-Asia Region (37.7%).

**Figure 1:**
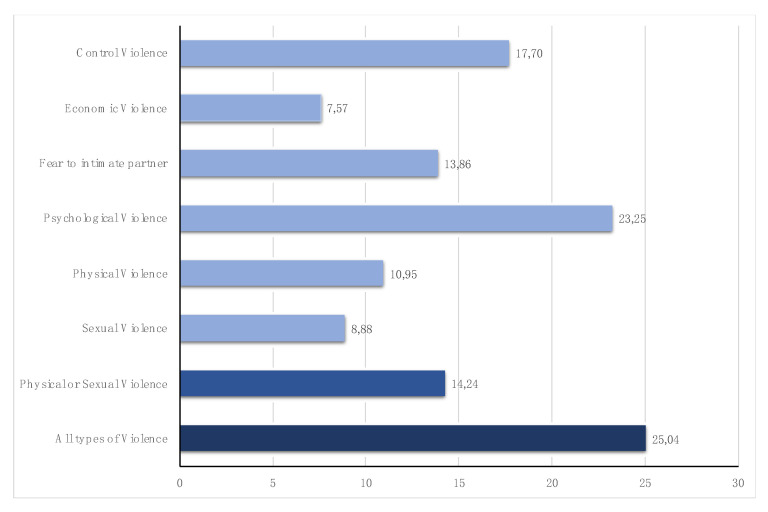
Incidence of different types of IVP against women in Spain. Source: VI Violence against Women Macro-survey-2019.

Data on Figure 1 show percentages similar to those for Spain in the FRA Gender-based violence against women survey [5], except in the case of sexual violence, which doubles the data provided by the European agency. In any case, the lower incidence of all types of violence compared to the European average is confirmed. Additionally, by the same token, data are lower than the data for the USA based on the National Intimate Partner and Sexual Violence Survey (NISVS) [29].

Regarding the type of violence, the most frequent is psychological violence (23.25%), which includes control violence (17.7%) and economic violence (7.57%). This data is lower than that recorded for the whole of Europe (43%) [30], although similar to that indicated by the authors for the Spanish case. The prevalence of physical or sexual violence stands at 14.24% of Spanish women. Even though the overall figure is similar to that estimated in other sources, the higher incidence of sexual violence is highlighted (8.88%), a figure that almost doubles the one shown by FRA [5].

The first objective of the study addresses the incidence of disability on IPV. For all types of violence, disability is a factor that increases the probability of experiencing IPV at values of just over 50%.

The case of economic violence is highlighted as a very relevant factor, doubling its incidence among women with disabilities. These data are consistent with the study of Kutin, Russell, and Reid [31]. This fact reflects a lower autonomy and greater control of their financial and heritage resources by the intimate partner, coercing their freedom and the possibility of escaping this abuse. Disability also increases the probability of suffering psychological, physical, or sexual violence by almost 50%. These results, in line with those obtained in Canada [32], USA [9], or Australia [31], indicate the greater vulnerability to IPV of women with disabilities, thus justifying the more detailed study of violence in this group.

This higher incidence is also reflected in the increased use of health services, as shown in Table 3. In addition, there are also higher percentages of women with disabilities who report physical and/or psychological consequences of IPV, or who indicate a significantly greater need for health and/or social services to mitigate these consequences, as shown in Table 4.

The information provided by the VWM-2019 shows the prevalence, in Spain, of the public health problem caused by violence against women by intimate partners, as well as the negative incidence of disability in this phenomenon. However, the survey, designed to measure violence against women globally, does not allow for a more in-depth analysis. Although IPV mainly affects women also affects men and non-binary people. Furthermore, an intersectional approach with other demographic variables (race, sexuality, ethnicity, religious beliefs, etc.) can help to interpret the incidence of IPV. However, these factors are not included in the VWM-2019, so the results of the study are limited by the availability of quantitative information.

Specifically, as it is not a health survey, aspects related to the quality of health care are not studied; nor does it delve into the causes of the need for such care, or the special needs of some groups, such as women with disabilities, the elderly, immigrants, or other vulnerable groups. This is a limitation for a more detailed study of how IPV affects women with different conditions. Therefore, the results of this work should be reviewed as more detailed information on the health aspects of IPV and on disability becomes available.

In summary, the prevalence of women suffering IPV in Spain reaches 25%. This percentage shows that it is a major problem that affects our society. Moreover, the prevalence is higher when it is a woman with a disability who experiences it, increasing from 24.30% to 32.20%.

## 5. Conclusions

Violence against women by intimate partners is a public health issue, with implications for both their health and the increase in use of health services. The problem of IPV is aggravated in the group of women with disabilities, with a higher incidence of all types of violence.

The association between IPV and use of health services has important implications for planning by health service providers. Therefore, IPV against women with disabilities is receiving more attention by international public health experts. The impact on the deterioration of physical and psychological health increases when a woman who is a victim of IPV has a disability, leading to greater use of health and social services.

Health service providers, and other organizations related to IPV, must be aware of the specific needs of women with disabilities and their differentiated use of health ser-vices. This knowledge will allow providers a greater training for the care of these women. Furthermore, given the higher economic and social cost of health care for women with disabilities who have suffered IPV, and from a public health perspective, the interaction between IPV and disability requires greater attention from the public administration, and it should justify the adoption of public policies and measures to combating such violence specifically focused on this group, which can both mitigate the consequences on women’s health and efficiently address a preventive approach to mitigate intimate partner violence.

## Figures and Tables

**Table 1:** Sociodemographic characteristics: Count, frequency, and percentage of women with Intimate Partner Violence (IPV).

Women with Disabilities	Count	Frequency	% With IPV
No	8630	90.20%	24.30%
Yes	939	9.80%	32.20%
**Age**	Count	Frequency	% With IPV
From 16 to 30 years old	1739	18.20%	28.60%
From 31 to 45 years old	2442	25.50%	31.70%
From 46 to 65 years old	3188	33.30%	24.6%
From 66 to 96 years old	2200	23.00%	15.50%
**Educational Level**	Count	Frequency	% With IPV
Less than Primary Education	127	2.00%	23.60%
Primary Education	1078	17.30%	17.90%
Secundary Education	3604	57.70%	25.60%
Higher Education	1440	23.00%	18.80%
**Labour Status**	Count	Frequency	% With IPV
Employed	4301	45.20%	29.40%
Unemployed	1357	14.30%	31.50%
Pensioner	2116	22.20%	18.10%
Student	592	6.20%	20.40%
Unpaid Domestic Labour	1150	12.10%	16.10%
**Nationality**	Count	Frequency	% With IPV
Spanish	8374	87.50%	23.20%
Spanish and other	448	4.70%	40.80%
Other	745	7.80%	36.50%
**Currently Having an Intimate Partner**	Count	Frequency	% With IPV
No	2705	29.40%	33.90%
Yes	6506	70.60%	22.70%
**Mother with Minor Children**	Count	Frequency	% With IPV
No	4119	60.60%	21.50%
Yes	2683	39.40%	29.90%
**Town Size**	Count	Frequency	% With IPV
Less than 10,000 inhabitants	1874	19.60%	20.90%
From 10,000 to 100,000 inhabitants	3737	39.10%	25.70%
From 100,000 to 400,000 inhabitants	2137	22.30%	26.50%
More than 400,000 inhabitants	1820	19.00%	26.10%

Source: Own elaboration from VI Violence against Women Macro-survey 2019 data.

**Table 2:** Incidence of IPV experienced by women with disabilities.

	Without Disabilities	With Disabilities	F-Statistic (Sig.)	OR (95% CI)
Control violence	17.1%	23.3%	22.852 (<0.001)	1.478 (1.258–1.737)
Economic violence	7.0%	13.1%	45.293 (<0.001))	2.008 (1.632–2.470)
Psychological violence	22.5%	30.1%	26.943 (<0.001)	1.478 (1.274–1.715)
Physical violence	10.4%	16.1%	28.550 (<0.001)	1.657 (1.374–1.999)
Sexual violence	8.4%	12.8%	19.488 (<0.001)	1.585 (1.289–1.948)
Fear to intimate partner	13.4%	18.2%	17.053 (<0.001)	1.448 (1.214–1.728)
All types of violence	24.3%	32.2%	28.056 (<0.001)	1.478 (1.278–1.710)

Source: Own elaboration from VI Violence against Women Macro-survey 2019 data.

**Table 3:** Incidence of disability on women’s health and the use of healthcare services.

	Without Disability	With Disability	F-Statistic (Sig.)	OR (95% CI)
Staying in bed at least one day for health reasons	56.4%	62.6%	3.158 (0.076)	1.290 (0.974–1.708)
Visit to a healthcare center or general practitioner	91.2%	94.4%	3.221 (0.073)	1.672 (0.949–2.946)
Admission to a hospital	28.1%	34.2%	3.806 (0.051)	1.339 (0.998–1.797)
Use of emergency services	52.5%	66.2%	16.118 (<0.001)	1.781 (1.339–2.368)
Visit to a psychologist, psychotherapist, or psychiatrist	23.1%	36.4%	18.327 (<0.001)	1.899 (1.409–2.560)
Consumption of tranquilizers	34.9%	49.8%	19,655 (<0.001)	1.860 (1.408–2.457)
Consumption of antidepressants	26.3%	38.2%	14.016 (<0.001)	1.735 (1.296–2.323)
Consumption of analgesics	80.7%	76.8%	1.911 (0.167)	0.791 (0.568–1.103)

Source: Own elaboration from VI Violence against Women Macro-survey 2019 data.

**Table 4:** Incidence of disability on IPV consequences.

	Without Disabilities	With Disabilities	F-Statistic (Sig.)	OR (95% CI)
Physical consequences due to IPV	25.7%	32.2%	5.734 (0.017)	1.374 (1.058–1.783)
Need for healthcare services due to IPV	34.9%	46.2%	8.761 (0.003)	1.597 (1.168–2.183)
Psychological consequences due to IPV	69.3%	74.4%	3.218 (0.073)	1.285 (0.976–1.692)
Medicine use due to IPV	19.4%	31.8%	24.224 (<0.001)	1.926 (1.477–2.512)
Alcohol use due to IPV	4.6%	3.3%	0.814 (0.377)	0.741 (0.386–1.423)
Hard drugs use due to IPV	2.4%	2.6%	0.060 (0.807)	1.099 (0.515–2.348)
Access to social services due to IPV	13.8%	20.2%	8.439 (0.004)	1.573 (1.156–2.140)

Source: Own elaboration from VI Violence against Women Macro-survey 2019 data.

## Data Availability

Not applicable.
